# The Correct Thing

**Published:** 1900-07

**Authors:** 


THE CORRECT THING.—The Colorado county democracy, in their recent "primary," inserted the following in their resolutions. If the State convention would take some action in the matter the thing could be done:

6. That in view of the wide spread prevalence of smallpox over the State, the ravages of meningitis and the destructive work of other preventable diseases during the last three years, our senator and representatives in the next legislature be instructed to exert themselves to secure such revision of health laws as will place the State on a parity with other States in the Union in sanitary matters.

---

**A SERIOUS FARCE.**

DR. S. C. RED, of Houston, well known to the readers of the JOURNAL as one of the most earnest workers in the cause of legitimate medicine and reform in our methods of licensing physicians—a member of the legislative committee, writes the JOURNAL a letter (see correspondence), in which he cites an occurrence illustrating the defect in the system of licensing, and the absurdity of the law as it now stands. The District Medical Examining Boards in some sections of Texas are an open door to quackery. It is the fault of the law, and, unless they are corrupt—not the fault of the boards. The boards have a right to fix and presumably do fix the standard, and if it is so low that *anybody* can pass—which is the case with some boards—where the passing means fifteen dollars—no one can say them nay. Members of these boards are appointed by the district judges,—and they have no means of knowing the qualifications or the character of their appointees, and I suppose they do not want to know; they are political, and every appointment means votes for the judge; it is a lever,—it is *"patronage,"* which he dispenses where it will do (him) most good. That a board should pass a batch of first course students (at $15 each), is not at all surprising; the board is the sole judge of the students' fitness to practice medicine and we have no right to impugn their motive in granting certificates; it is the fault of the law that such things are possible. The law should be repealed, and in its stead one passed creating a State Board of Medical Examiners, composed of physicians selected for their known probity and professional qualifications. We have written on this and similar evils till the subject seems worn to a frazzle; but the only way to get a reform is to keep pegging away; hence we "peg." But we again confess that we have little hope of success. Drs. Harrison, Red, Saunders and Smith are the Committee on State Board of Health, and the profession are deeply interested in their efforts to "take these matters out of politics."

